# Identification and validation of hub genes expressed in ulcerative colitis with metabolic dysfunction-associated steatotic liver disease

**DOI:** 10.3389/fimmu.2024.1357632

**Published:** 2024-03-14

**Authors:** Yupei Liu, Jiao Li, Shan Tian, Qingzhi Lan, Zhiyi Sun, Chuan Liu, Weiguo Dong

**Affiliations:** ^1^ Department of Gastroenterology, Renmin Hospital of Wuhan University, Wuhan, China; ^2^ Department of Infection, Union Hospital of Tongji Medical College of Huazhong University of Science and Technology, Wuhan, China; ^3^ Department of Pathology, Renmin Hospital of Wuhan University, Wuhan, China; ^4^ Department of Biostatistics, School of Public Health, University of Michigan, Ann Arbor, MI, United States

**Keywords:** ulcerative colitis, metabolic dysfunction-associated steatotic liver disease, M1 macrophages, immune, bioinformatics

## Abstract

**Objective:**

Ulcerative colitis (UC) and metabolic dysfunction-associated steatotic liver disease (MASLD) are closely intertwined; however, the precise molecular mechanisms governing their coexistence remain unclear.

**Methods:**

We obtained UC (GSE75214) and MASLD (GSE151158) datasets from the Gene Expression Omnibus (GEO) database. Differentially expressed genes (DEGs) were acquired by the ‘edgeR’ and ‘limma’ packages of R. We then performed functional enrichment analysis of common DEGs. Hub genes were selected using the cytoHubba plugin and validated using GSE87466 for UC and GSE33814 for MASLD. Immunohistochemistry was employed to validate the hub genes’ expression in clinical samples. Immune infiltration and gene set enrichment analyses of the hub genes were performed. Finally, we estimated the Spearman’s correlation coefficients for the clinical correlation of the core genes.

**Results:**

Within a cohort of 26 differentially regulated genes in both UC and MASLD, pathways involving cytokine-mediated signaling, cell chemotaxis, and leukocyte migration were enriched. After further validation, *CXCR4, THY1, CCL20*, and *CD2* were identified as the hub genes. Analysis of immune infiltration patterns highlighted an association between elevated pivotal gene expression and M1 macrophage activation. Immunohistochemical staining revealed widespread expression of pivotal genes in UC- and MASLD-affected tissues. Furthermore, significant correlations were observed between the increased expression of hub genes and biochemical markers, such as albumin and prothrombin time.

**Conclusion:**

This bioinformatics analysis highlights *CXCR4, THY1, CCL20*, and *CD2* as crucial genes involved in the co-occurrence of UC and MASLD, providing insights into the underlying mechanisms of these two conditions.

## 1 Introduction

Inflammatory bowel disease (IBD) is a chronic and recurrent inflammatory condition of uncertain origin that affects the gastrointestinal tract. As one subtype of IBD, ulcerative colitis (UC) is characterized by continuous inflammation of the colonic mucosa. Common symptoms include diarrhea, abdominal pain, passage of mucus and blood, and weight loss (1). In addition to the intestinal manifestations, UC is often associated with extraintestinal manifestations involving the hepatobiliary system, such as fatty liver and primary sclerosing cholangitis (2, 3). The etiology of UC is not yet fully understood; however, it is recognized as a multifactorial IBD. The global incidence and prevalence of UC is rapidly increasing, posing a significant challenge to global public health. Effective managing ulcerative colitis remains a major challenge (4).

Metabolic dysfunction-associated steatotic liver disease (MASLD), formerly known as nonalcoholic fatty liver disease (NAFLD), was officially renamed in June 2023 (5). MASLD is a pathological syndrome characterized by hepatic steatosis and lipid accumulation. Within the category of metabolic syndrome-related chronic liver diseases, the prevalence of MASLD is increasing at an alarming rate, accounting for 32.4% of the total population by 2022, and is one of the most common chronic liver diseases (6). As a common extra-intestinal manifestation of IBD, MASLD frequently coexists with IBD. It is estimated that up to 26.1% of patients with IBD have concurrent MASLD. Among the patients with MASLD, the prevalence of UC was as high as 41.7% (7).

Although increasing evidence suggests a close association between UC and MASLD, the mechanisms underlying the simultaneous occurrence of these two diseases are not fully understood. Moreover, the increasing prevalence of both diseases inevitably increases healthcare costs (8, 9). Therefore, it is imperative to explore the relationship between UC and MASLD in terms of comorbidity mechanisms. This study aimed to use bioinformatics in conjunction with clinical samples to identify diagnostic biomarkers and relevant signaling pathways for MASLD in the context of UC. These findings provide clinical evidence for the treatment of patients with UC complicated with MASLD.

## 2 Materials and methods

### 2.1 Data sources

**Figure 1:**
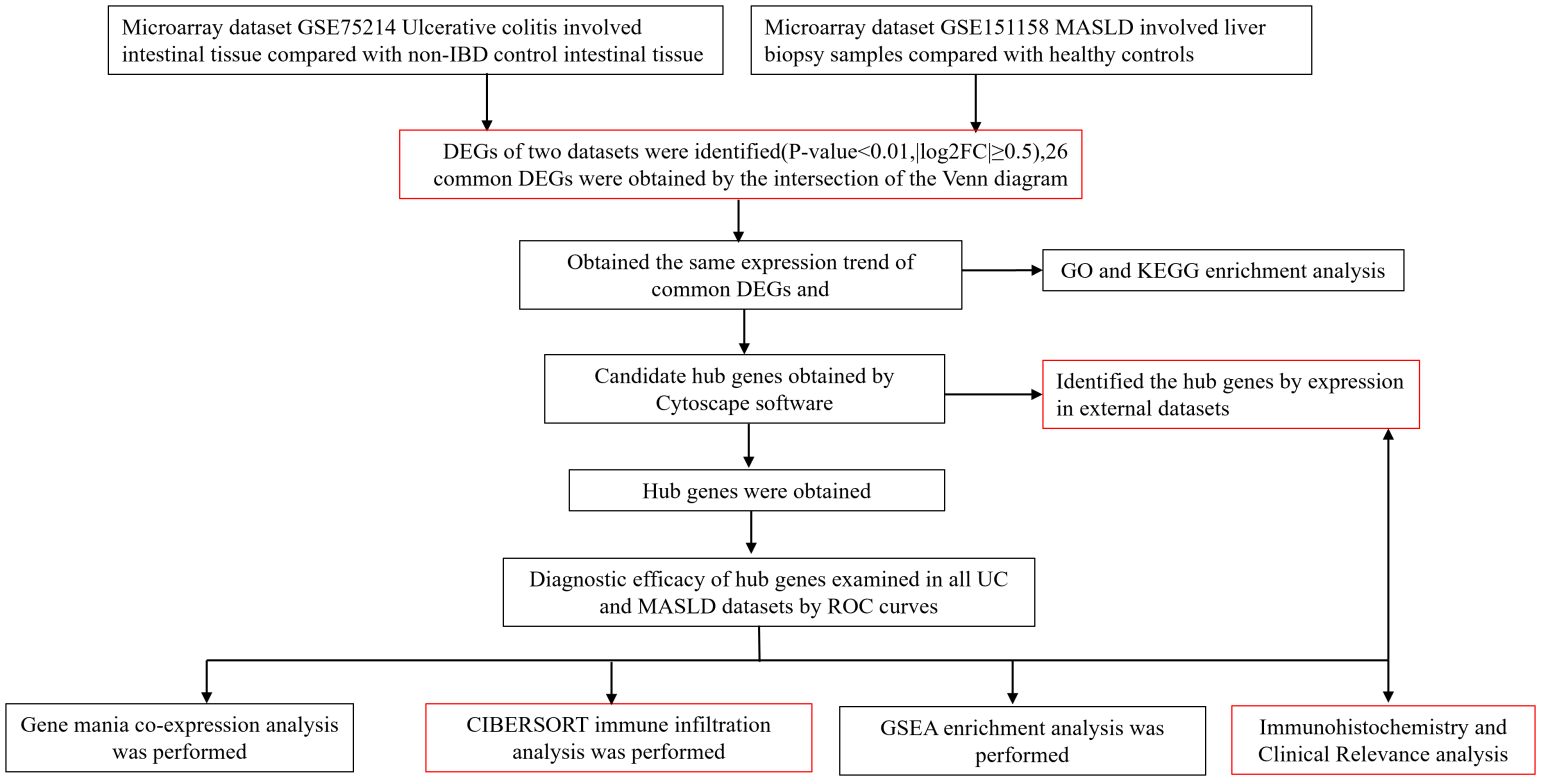
Roadmap of the main research ideas in this article. The steps highlighted in red are the innovative points or the necessary steps of our study.

Four data sets (GSE75214, GSE151158, GSE87466, GSE33814) were downloaded from the Gene Expression Omnibus (GEO) database (http://www.ncbi.nlm.nih.gov/geo). For the UC dataset, we selected GSE75214. This dataset included the RNA sequencing results from 97 patients with active UC and 11 normal control colon mucosa samples. The dataset has a large sample size, consistent sampling sites between the case and control groups, and provides gene expression profiles from mucosal biopsies, primarily from patients with active UC. It is considered a classic dataset for the bioinformatics analysis of UC. For the MASLD dataset, we selected the GSE151158. This dataset included sequencing results from 40 patients with MASLD and 21 normal control liver tissues. The dataset has a relatively large sample size and was recently published in the context of this disease. Gene analysis of this dataset mainly focused on genes involved in innate immune function, making it more suitable for investigating the comorbidity mechanisms between MASLD and UC, an autoimmune disease (10, 11). The validation datasets used were GSE87466 and GSE33814. GSE87466 contained data from 87 patients with UC, whereas GSE33814 contained data from 31 patients with MASLD. These validation datasets also have the advantages of relatively large sample sizes and recent publication dates. They have been extensively reported in the relevant literature, and their analytical results are considered authoritative and reliable (12, 13). A comprehensive insight into the datasets used in this study is outlined in **Supplementary Table 1**. **Supplementary Table 2** provides the comparability of the GEO and hospital cohorts in terms of age and sex. The roadmap of the main research ideas in this article is illustrated in **Figure 1**.

### 2.2 Definitions

UC patients: All patients with UC included in this study had a definitive diagnosis supported by relevant endoscopic and pathological evidence. In addition, patients were required to be over 18 years of age and possess complete hospitalization records and laboratory test results. The exclusion criteria included patients with incomplete clinical or pathological information, those with an unclear diagnosis, and those with concurrent tumors or severe complications.

Patients with MASLD: MASLD was clinically diagnosed based on meeting ultrasound or imaging-based criteria for the condition. Exclusion in the absence of other causes of steatosis such as significant alcohol consumption, chronic viral hepatitis B or C and other specific causes for hepatitis steatosis, the presence of at least one of the following five metabolic abnormalities is required (1): Overweight or obesity: BMl≥25 kg/m^2^ in Caucasians (BMl≥23 kg/m^2^ in Asians), or waist circumference>94/80 cm in Caucasian men and women, or ethnic adjusted criteria (2). Fasting glucose >5.6 mmol/L (100 mg/dL), or 2-hour post-load glucose ≥7.8 mmol/L (140 mg/dL), or hemoglobin A1c≥39 mmol/mol (5.7%), or type 2 diabetes mellitus or treatment for type 2 diabetes (3). Blood pressure≥130/85 mmHg or specific drug treatment. (4) Plasma triglycerides >1.70 mmol/L (150 mg/dL) or lipid-lowering treatment. (5) Plasma HDL-cholesterol <1.0 mmol/L (40 mg/dL) for men and <1.3 mmol/L (50 mg/dL) for women or lipid-lowering treatment (5).

Ten healthy controls and 49 patients with UC were included in this study. Seven patients had concurrent MASLD. **Supplementary Table 3** provides clinical information of all patients included in the study. All clinical data and intestinal tissue samples were obtained from patients treated at the Renmin Hospital of Wuhan University between 2021 and 2023.

### 2.3 Analysis of differential genes

The microarray data were processed via two R packages, “GEOquery” and “Limma”. Probe sets devoid of the corresponding gene symbols were excluded from the analysis. In cases where genes were associated with multiple probe sets, the average was calculated. Differentially expressed genes (DEGs) were identified based on the criteria of p-values < 0.01 and |fold change| > 1. To identify co-occurring DEGs shared by the UC and MASLD datasets, Venn diagrams were generated using the online tool Evenn (http://www.ehbio.com/test/venn/#/) (14).

### 2.4 Functional enrichment analysis

To investigate the intrinsic molecular biological mechanisms associated with these co-occurring genes, Gene Ontology (GO) and Kyoto Encyclopedia of Genes and Genomes (KEGG) enrichment analyses were performed (15, 16) using the ‘ClusterProfiler’ package (version 4.8.2) in R (17). GO and KEGG serve as pivotal public repositories for delineating the distinctive biological and functional traits within these pathways.

### 2.5 Protein-protein interaction network construction and module analysis

The online resource STRING (http://string-db.org) was used to probe interconnected genes (18). The protein-protein interaction (PPI) networks featuring multifaceted regulatory connections were constructed using the STRING database. Interactions with a composite score surpassing 0.4 were deemed statistically significant. PPI networks were visualized using Cytoscape (http://www.cytoscape.org) (19). Notably, the Cytoscape Molecular Complex Detection Technology (MCODE) is a technique that clusters proteins based on their relationships with edges and nodes. It aims to identify key subnetworks and protein complexes within the biological molecular interaction network, leveraged for the identification of principal functional modules in PPI networks. The criteria for module selection were Degree Cutoff = 2, Node Score Cutoff = 0.2, K-Core = 2, and Max. Depth = 100.

### 2.6 Identification and validation of hub gene expression

Hub genes were identified using the CytoHubba plugin of Cytoscape. Four algorithms were employed to analyze the co-expressed genes, and the top 15 comorbid genes from each algorithm were retained and intersected to obtain the candidate core genes. Subsequently, based on their expression in the validation datasets GSE87466 and GSE33814, the candidate core genes were further refined and identified as the core genes for comorbidity between UC and MASLD.

### 2.7 Construction of receiver-operating characteristic curves to assess diagnostic efficacy

The ‘pROC’ package was utilized to generate the receiver operating characteristic (ROC) curve, evaluating the ability of hub genes to differentiate between UC patients and healthy individuals across all datasets (20).

### 2.8 Exploration the internal associations and immune cell correlations of hub genes

For the establishment of co-expression networks encompassing these hub genes, the GeneMANIA platform (http://www.genemania.org/) was utilized (21), which is commonly employed to uncover the internal associations within gene sets. The correlation between varying degrees of immune cell infiltration and diagnostic markers for MASLD comorbidity in UC was investigated through the CIBERSORT (https://cibersortx.stanford.edu/) method for immune infiltration analysis using the R package ‘CIBERSORT’ (22). Spearman’s correlation analysis was subsequently employed to examine the relationship between the expression of diagnostic markers and the content of 22 distinct immune cell types to elucidate the potential associations between diagnostic markers and immune cell populations.

### 2.9 Gene set enrichment analysis

Gene Set Enrichment Analysis (GSEA) was used to evaluate pertinent pathways and molecular mechanisms distinguishing the two study groups (23). We downloaded gene expression data for each sample from the GEO database, and based on the median expression of the core genes in the chip, we re-grouped the samples into high and low expression groups. After using signal-to-noise measurement in GSEA to rank all genes in the chip according to their association with the high or low expression of the core genes, we then selected the top 5 pathways associated with high expression of each core gene. Gene sets showing enrichment with a nominal p-value of < 0.05, |normalized enrichment score (NES)|> 1, and a false positive rate (FDR) q-value < 0.25 were classified as statistically significant.

### 2.10 Immunohistochemical staining

Immunohistochemistry was used to assess the expression of *CXCR4, CCL20, THY1*, and *CD2* in the intestinal tissues of the patients at the Department of Pathology, Renmin Hospital of Wuhan University. Paraffin sections (5 μm) were soaked in 3% hydrogen peroxide for 15 min after deparaffinization with xylene and hydration with graded ethanol. Sections were then blocked with goat serum for 0.5 h at room temperature. Subsequently, sections were incubated overnight at 4°C with primary antibodies against *CXCR, CCL20, THY1*, and *CD2* (Abcam). The following day, samples were tested using an immunohistochemistry kit (D601037, Sangon Biotech, Shanghai, China). Sections were incubated with HRP-conjugated secondary antibody for 0.5 h at 37°C and subsequently visualized with DAB chromogen solution. Nuclei were counterstained with hematoxylin. Finally, microscopy (Olympus FV1200, Japan) was used for image acquisition, and the percentage area of positive staining was determined using K-reviewer software.

The results of immunohistochemical staining based on the Immunoreactive Score (IRS) were primarily evaluated by two pathology researchers. Distinct components, such as the extent of staining (0-3 points) and positivity rate (0-4 points) within the immunohistochemical sections, were assessed separately and subsequently multiplied to yield a comprehensive score ranging from 0 to 12 points. Specifically, staining intensity was evaluated based on the appearance of staining in the target cells: no staining (0 points), pale yellow (1 point), brownish yellow (2 points), and tan (3 points). Similarly, the percentage of positive cells was assessed in terms of the positivity rate: 0%-5% (0 points), 6%-25% (1 point), 26%-50% (2 points), 51%-75% (3 points), and >75% (4 points). The amalgamated score was categorized into high and low positivity and was subsequently harmonized.

Considering the potential selection bias, the histological images shown in this manuscript represent patients with UC and moderate disease activity. The study complied with the Declaration of Helsinki and was approved by the Ethics Committee Review Board of Renmin Hospital of Wuhan University. A consent form was sent to and signed by a legal custodian. The Institutional Review Board of Renmin Hospital of Wuhan University approved the study protocol. (Approval number: WDRY2022-K130) A nonparametric Wilcoxon signed-rank test was employed to assess differences between the groups, while analysis of variance (ANOVA) was utilized for comparing data across multiple groups. A p value < 0.05 was considered significant (*p < 0.05, **p < 0.01, ***p < 0.001, ****p < 0.0001).

### 2.11 Correlation of hub genes with disease characteristics and biochemical levels in UC

As previously described, to further elucidate the association between core genes and diseases, we performed a correlation analysis between the clinical information of patients with UC and the IRS scores of the core genes. A p value < 0.05 was considered significant, an r value > 0.3 indicated a certain degree of correlation, and an r value > 0.5 indicated a strong correlation.

### 2.12 Statistical analysis

All statistical analyses of this bioinformatics study were conducted using R software (version 4.3.1; https://www.r-project.org/). The two datasets were compared using Wilcoxon signed-rank tests. Correlation analyses were performed using Spearman’s correlation coefficients. Statistical significance was set at p < 0.05.

## 3 Results

### 3.1 Identification of differentially expressed genes in the expression profiles of UC and MASLD

**Figure 2:**
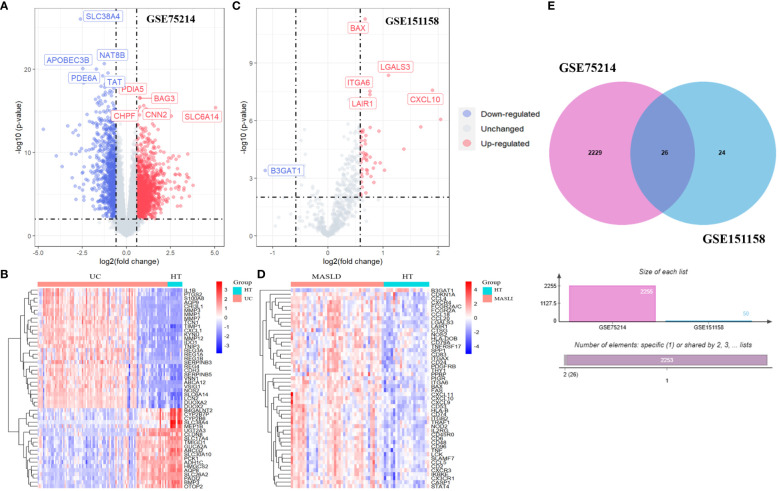
The volcano map **(A)** and heatmap **(B)** of GSE75214; The volcano map **(C)** and heatmap **(D)** of GSE151158. **(E)** The Venn diagram of 26 DEGs was obtained by overlapping two datasets. DEG, differentially expressed gene.

Volcano and heat maps demonstrated that 2,255 differential genes were identified in 97 patients with UC and 11 normal controls from GEO, of which 1,302 were upregulated and 953 were downregulated genes. *SLC38A4, NAT8B, APOBEC3B, PDE6A*, and *TAT* are the most significantly downregulated genes, while *PDIA5, BAG3, CHPF, CNN2*, and *SLC6A14* are the most significantly upregulated genes (**Figure 2A**). A heatmap of the 50 most remarkable DEGs in the UC dataset is shown in **Figure 2B**. In 40 patients with MASLD and 21 normal controls, 50 differential genes were identified, of which 49 were upregulated and 1 was downregulated. The only downregulated gene is *B3GAT1*, while *BAX, ITGA6, LAIR1, LGALS3*, and *CXCL10* are the most significantly upregulated genes (**Figure 2C**). A heat map of the top 50 most prominent DEGs in the MASLD dataset is shown in **Figure 2D**. Using a Venn diagram, the intersection of the differential gene sets from the two datasets led to the identification of 26 co-occurring differentially expressed genes. Notably, all 26 genes were up-regulated (**Figure 2E**).

### 3.2 Functional characterization of common differentially expressed genes

**Figure 3:**
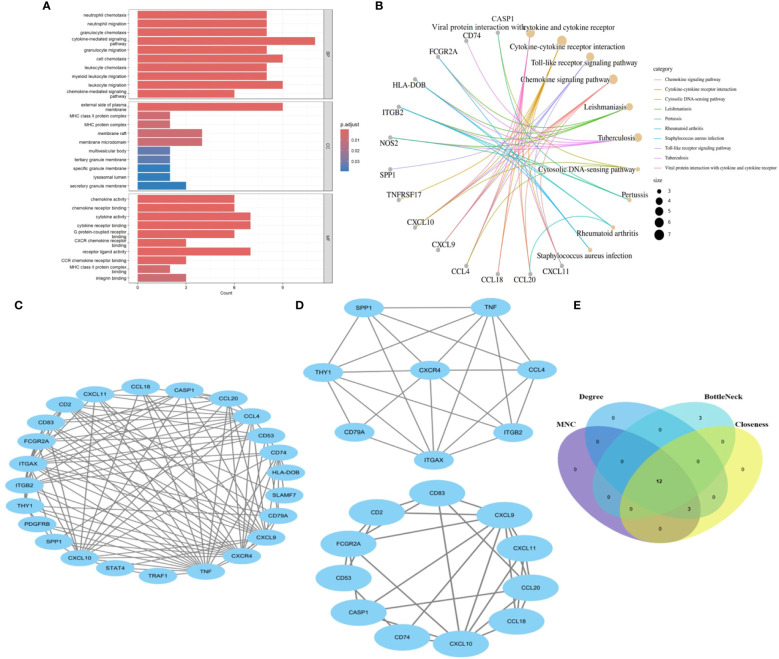
**(A)** The bar diagram of top 10 items in BP, CC and MF of GO enrichment analysis. **(B)** The concept map of top 10 pathways in KEGG enrichment analysis. **(C)** PPI network diagram. **(D)** Two significant gene clustering modules. **(E)** Identification of 12 candidates for hub genes by four algorithms. BP: Biological Process; CC, Cellular Component; MF, Molecular Function; GO, Gene Ontology; KEGG, Kyoto Encyclopedia of Genes and Genomes. PPI, protein–protein interaction;.

We identify the underlying mechanisms of shared genes related to MASLD in the progression of UC. Using the shared 26 DEGs observed in both UC and MASLD (**Figure 3A**), our analysis yielded 189 distinct GO terms including 166 biological processes (BPs), 12 molecular functions (MFs), and 11 cellular components (CCs). With reference to BP, the core genes were mostly enriched in neutrophil chemotaxis (GO:0030593), neutrophil migration (GO:1990266), and granulocyte chemotaxis (GO:0071621). Additionally, for molecular functions (MF), the core genes showed prominent enrichment in chemokine activity (GO:0008009), chemokine receptor binding (GO:0042379), and cytokine activity (GO:0005125). With reference to CC, the core genes were mainly enriched in the outer plasma membrane (GO:0009897), MHC class II protein complexes (GO:0042613), and MHC protein complexes (GO:0042611). In addition, the results of our KEGG enrichment analysis showed that the core genes were enriched in 89 pathways (**Figure 3B**), among which viral protein interaction with cytokines and cytokine receptor (hsa04061, P=6.738e-06), cytokine-cytokine receptor interaction (hsa04060, P=7.978e-05), Toll-like receptor signaling pathway (hsa04620, P=7.978e-05), and Chemokine signaling pathway (hsa04062, P=7.978e-05) were significantly associated with enrichment of core genes. This confirms the presence of shared molecular mechanisms between UC and MASLD, indicating the intricate connections underlying the two conditions.

### 3.3 Key genes of the PPI network and identification of hub genes

To reveal the interactions between proteins, a PPI network of the shared genes was constructed using the STRING database and Cytoscape software (**Figure 3C**). Twenty-four nodes and 112 pairs were obtained (p< 1.0e-16). MCODE, a Cytoscape plugin, was employed for modular analysis to detect key clustering modules. Utilizing the MCODE plugin, significant modules in the PPI network were uncovered, identifying 19 hub genes tightly connected in two clusters as crucial modules (**Figure 3D**), Module 1 comprised 8 nodes and 22 edges with a cluster score of 6.286, while Module 2 consisted of 11 nodes and 28 edges with a cluster score of 5.6. Additionally, hub genes related to common DEGs were pinpointed using CytoHubba. As biological networks are heterogeneous, multiple topological analysis algorithms have been used simultaneously to identify hub genes (24). maximum neighborhood component (MNC), Degree, BottleNeck and Closeness were simultaneously applied to forecast and investigate the top 15 important hub genes in the PPI networks. The overlap of 15 genes from these four algorithms unveiled 12 potential hub genes. (**Figure 3E**): *CCL20, CXCL9, CD2, CXCR4, TNF, CD74, CCL4, CD53, THY1, ITGAX, CXCL10*, and *CD83*.

### 3.4 Validation of hub genes expression

**Figure 4:**
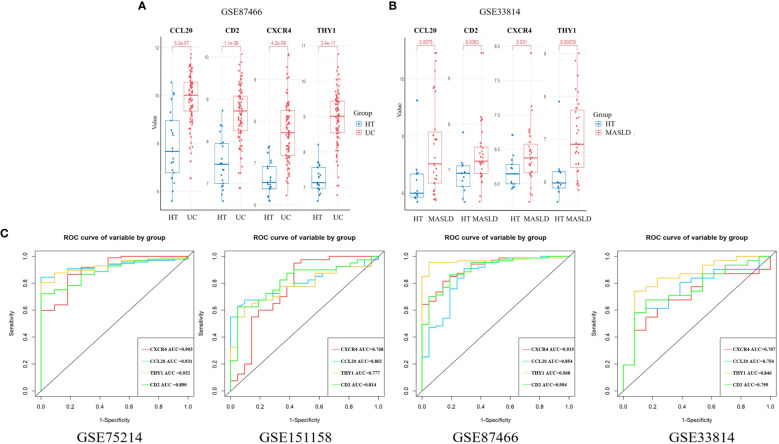
The expression of hub genes in the validation sets. **(A)** CXCR4, CCL20, THY1, and CD2 were validated in GSE 87466. **(B)** CXCR4, CCL20, THY1, and CD2 were validated in GSE33814. **(C)** ROC curves were plotted to assess the diagnostic accuracy of the hub gene. UC, ulcerative colitis; MASLD, metabolic dysfunction-associated steatotic liver disease; ROC, receiver operating characteristic.

We chose the UC dataset GSE87466 and MASLD dataset GSE33814 from GEO to verify the expression of the candidate hub genes. The findings revealed that only four hub genes exhibited significant upregulation in both UC and MASLD tissues compared to those in healthy tissues (**Supplementary Figure 1**). These hub genes were *CXCR4, CCL20, THY1*, and *CD2* (**Figures 4A, B**).

### 3.5 Using ROC curve to evaluate diagnostic efficacy in UC and MASLD

ROC curves for the four hub genes were generated and AUCs (Area Under Curve) were determined. In the UC training set, AUCs for *CXCR4, CCL20, THY1*, and *CD2* hub genes were 0.903, 0.931, 0.932, and 0.890, respectively. In the MASLD training set, the AUCs of the hub genes were 0.768, 0.802, 0.777, and 0.814, respectively. In addition, the AUC values of hub genes exceeded 0.7 in validation datasets including GSE87466 and GSE33814, indicating the excellent predictive potential of these four hub genes (**Figure 4C**). These results indicate that *CXCR4, CCL20, THY1*, and *CD2* are promising markers for the diagnosis of UC and MASLD.

### 3.6 Analysis of core gene function and immune infiltration

**Figure 5:**
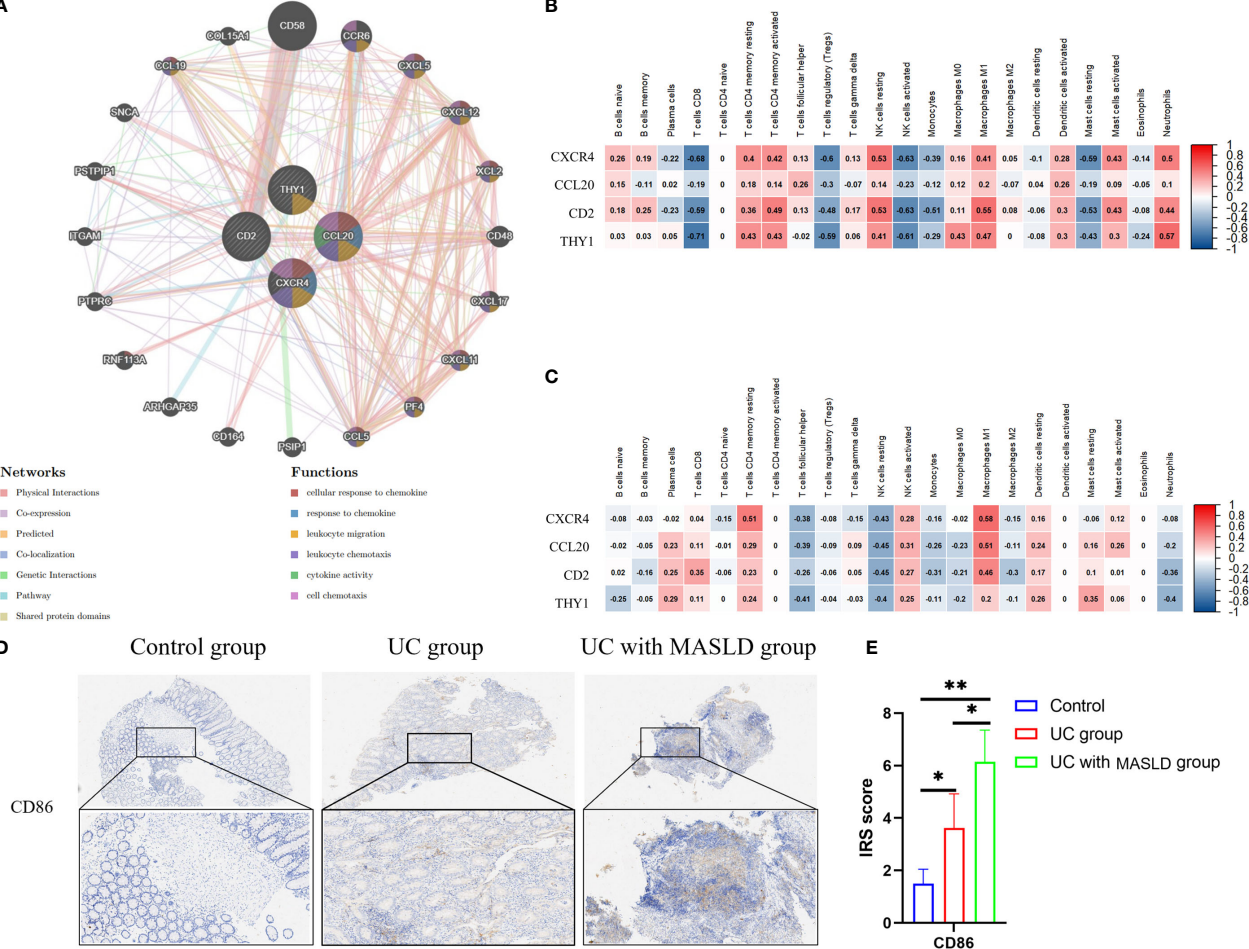
**(A)** The protein–protein interaction network generated from the GenMINIA database. **(B)** The correlation between hub genes and differential immune cells in GSE75214.Positive correlation is shown in red; Negative correlation is shown in blue. **(C)** The correlation between hub genes and differential immune cells in GSE151158. **(D)** CD86 expression in various groups of tissue samples. **(E)** IRS scores of CD86 among different groups. DEG: differentially expressed gene; IRS, Immunoreactive Score. *:P<0.05; **:P<0.01; ***P<0.001.

To investigate potential correlations and relationships, we performed a PPI analysis using the GeneMANIA database to examine the relationships between the four hub genes and their 20 interacting genes. This analysis aimed to predict correlations related to colocalization, shared protein structural domains, coexpression, prediction, and pathways. The predicted genes are depicted in the outer circle, while the hub genes are represented in the inner circle. As shown in **Figure 5A**, the resulting network exhibited enrichment in cellular responses to chemokines, chemokine responses, leukocyte migration, leukocyte chemotaxis, cytokine activity, and cellular chemotaxis.

To further explore the relationship between core genes and immune infiltration, the proportions of 22 immune cells in the GSE75214 and GSE151158 datasets were evaluated using CIBERSORT. Spearman analysis was subsequently performed to explore the correlation between the four hub genes and immune cells in UC and MASLD. In UC samples (**Figure 5B**), a positive relationship was observed between core genes and NK cells resting (r=0.53, p<0.05), M1 macrophages (r=0.55, p<0.05), neutrophils (r=0.57, p<0.05), and T cells CD4 memory activated (r=0.49, p<0.05). Conversely, in MASLD samples (**Figure 5C**), the core genes were primarily associated with M1 macrophages (r=0.59, p<0.05) and T cells CD4 memory resting (r=0.61, p<0.05). Taken together, the core genes associated with the comorbidity of UC and MASLD appear to be primarily correlated with the activation of M1 macrophages. To further investigate our hypothesis, we performed immunohistochemical staining of intestinal tissue samples to label *CD86* molecules that were highly expressed by M1 macrophages, and examined their expression across different groups (**Figure 5D**). The results revealed a significantly higher IRS score for *CD86* in the comorbidity group compared to both the UC and control groups (**Figure 5E**), providing additional confirmation of the role of M1 macrophages in the comorbidity of UC and MASLD.

### 3.7 GSEA results for hub genes

To identify the regulatory pathways that were differentially expressed between the high- and low-expression groups of hub genes within GSE75214 and GSE151158, we conducted GSEA and identified five pivotal signaling pathways that were activated in UC and MASLD based on their enrichment scores (**Supplementary Figures 2A, B**). The results revealed that the heightened expression of central comorbid genes in UC and MASLD was prominently linked to graft-versus-host disease, primary immunodeficiency, and allograft rejection. Additionally, in UC samples, elevated expression of these central genes was associated with leishmaniasis and malaria, whereas in MASLD samples, it was correlated with Type I diabetes mellitus and aspergillosis. The nominal p-values, FDR q-values, and NES of GSE75214 and GSE151158 are shown in **Supplementary Tables 4**, **5**. GSEA results for the validation datasets GSE87466 and GSE33814 are shown in **Supplementary Figures 3A, B**.

### 3.8 Validation of hub genes in tissue samples and evaluation of clinical relevance

**Figure 6:**
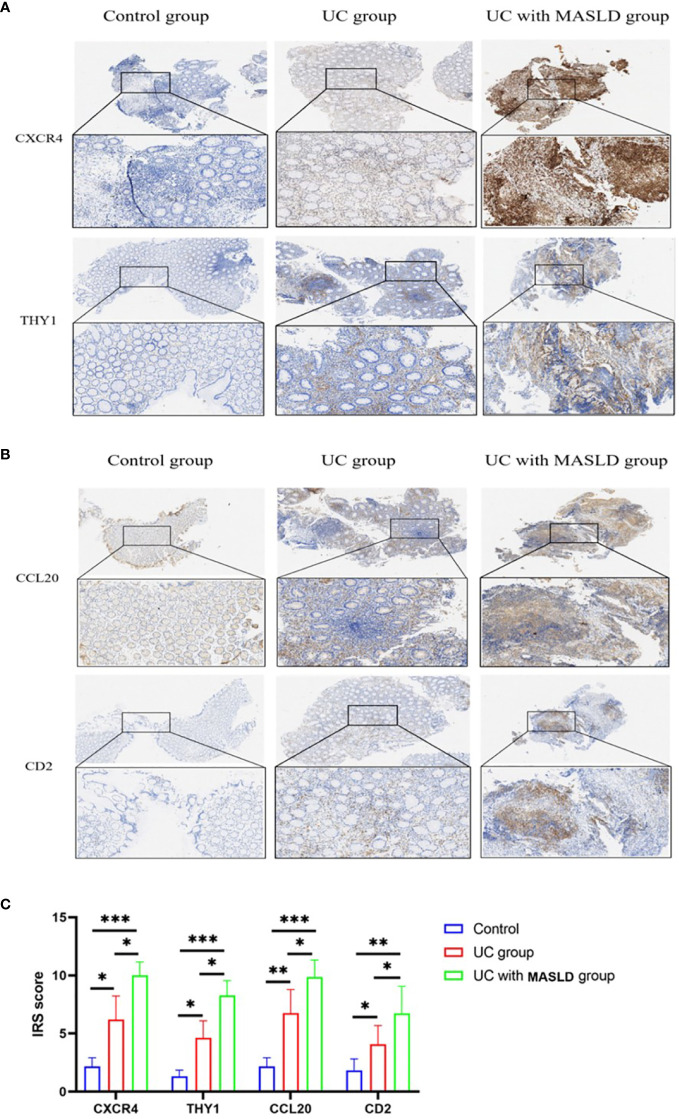
**(A)** Representative immunohistochemistry images of CXCR4 and THY1 expression in control, UC and UC-MASLD patient colon tissues. **(B)** Representative immunohistochemistry images of CCL20 and CD2 expression in control, UC and UC-MASLD patient colon tissues. **(C)** IRS of hub genes among different groups. UC, ulcerative colitis; MASLD, metabolic dysfunction-associated steatotic liver disease; IRS, Immunoreactive Score. *:P<0.05; **:P<0.01; ***P<0.001.

To further validate our hypothesis, we performed immunohistochemical staining of intestinal tissue sections obtained from patients diagnosed with UC, and standardized the expression levels of core genes using the IRS system. **Supplementary Figure 4** displays immunohistochemical images (4x) of core genes in patients with UC with varying degrees of disease activity. The Kruskal-Wallis test was conducted to compare the IRS scores of these core genes, and the results are presented in **Supplementary Table 6**, revealing no statistically significant differences in gene expression among the different UC disease severity groups. These findings could be applied to subsequent analyses of patients with coexisting MASLD. However, to minimize potential selection bias during the validation process and ensure the rigor of our study, tissue samples exclusively derived from intestinal tissue slices of patients with moderate UC were used (**Figures 6A, B**). Based on the IRS scores, the hub genes *CXCR4, THY1, CCL20*, and *CD2* exhibited low or negligible expression in normal tissues, with IRS scores of 2, 1.3, 2, and 1.5, respectively. In contrast, moderate expression was observed in the UC group, with scores of 6, 4.6, 6.7, and 4, and the expression levels were significantly higher in tissues from individuals with UC combined with MASLD, as evidenced by IRS scores of 10, 8.2, 9.8 and 6.7 (**Figure 6C**).

Furthermore, based on the clinical information collected from 42 patients with UC, we aimed to determine the clinical significance of these core genes. By adding the IRS scores of the four hub genes and calculating the total scores of hub genes for each patient with UC, we performed a Spearman correlation analysis between the total scores of hub genes and clinical information. The results indicate that the expression of core genes is closely associated with protein, electrolyte, coagulation function, and hemoglobin-related indicators in UC patients. The expression of core genes showed a significantly negative correlation with the levels of total protein (r=-0.326, p=0.035), albumin (r=-0.482, p=0.001), cholinesterase (r=-0.373, p=0.015), calcium concentration (r=-0.353, p=0.022), hemoglobin (r=-0.353, p=0.022), platelet distribution width (r=-0.313, p=0.044), and macro platelet % (r=-0.323, p=0.037), and positively correlated with the levels of urea/creatinine ratio (r= 0.464, p=0.002), blood magnesium concentration (r=0.335, p=0.030), prothrombin time (r=0.434, p=0.004), and PT international normalized ratio (r=0.442, p=0.003) (**Table 1**). Some of the biochemical indices that were significantly correlated with core genes are shown in **Supplementary Figure 5**.

**Table 1:** Correlation of total core gene score with biochemical parameters in patients with UC.

Variables	IRS score	
	r	P
Total Protein	-0.326	0.035
Albumin	-0.482	0.001
Cholinesterase	-0.373	0.015
Urea/Creatinine Ratio	0.464	0.002
Calcium	-0.353	0.022
Magnesium	0.335	0.030
Hemoglobin	-0.353	0.022
Platelet distribution width	-0.313	0.044
Macro platelet %	-0.323	0.037
Prothrombin time	0.434	0.004
INR	0.442	0.003

UC: ulcerative colitis; INR, PT International Normalized Ratio

## 4 Discussion

This study explored common hub genes and pathways associated with the co-occurrence of UC and MASLD using bioinformatics analyses of publicly accessible databases. We identified *CXCR4, CD2, THY1*, and *CCL20* as being significantly upregulated in patients diagnosed with UC together with MASLD, marking them as pivotal core genes. Immune infiltration analysis revealed a notable association between the increased expression of these core genes and the activation of M1 macrophages. This observation was further reinforced by the GSEA results, which demonstrated a significant link between elevated core gene expression and other immune-related diseases and pathways. MASLD is a common comorbidity of UC, potentially heightening the susceptibility of patients with IBD to liver-related complications compared with those without MASLD, which may affect the clinical management of patients with IBD (25).

The receptor *CXCR4* belongs to the chemokine receptor family and plays a critical role in cell signaling and immune regulation. Upon binding to its ligand *CXCL12, CXCR4* participates in numerous biological processes including cell migration, proliferation, survival, and tissue development (26). Previous studies identified *CXCR4* as an effective leukocyte chemoattractant in the development of UC. This process involves attracting lymphocytes and monocytes from the bloodstream to the site of inflammation, exacerbating colonic inflammation, and promoting the onset and progression of UC (27).

In non-alcoholic steatohepatitis (NASH) animal experiments, it has been demonstrated that *CXCR4/CXCL12* facilitates CD4T cell-dependent migration to the liver, promoting the recruitment of hepatic lymphocytes and the progression of MASLD (28). Furthermore, patients with NASH display specific lipidomic characteristics (29), especially elevated free cholesterol levels, which significantly affect the chemokine binding, conformational integrity, and functionality of *CXCR4* (30). This further enhances T-cell activation and their participation in intracellular signaling and inflammatory processes, thereby exacerbating steatohepatitis (31, 32). These findings imply that *CXCR4* is a potential therapeutic target for the treatment of UC-associated MASLD.

As a member of the chemokine ligand family, *CCL20* serves as both an inflammatory chemoattractant and a homeostatic chemokine (33). It contributes to the balanced development and maintenance of the mucosal immune system by continuously expressing follicle-associated epithelial cells in Peyer’s patches and isolated lymphoid follicles, thereby contributing to homeostasis (34). Specifically, *CCL20* can transition from a regulatory to a proinflammatory 1 type or 17 type response, promoting Th17 cell activation, increasing IL-17 secretion, and exacerbating inflammatory reactions (35). Studies have indicated that during the active phase of IBD development, the expression of *CCL20* mRNA increases several-fold owing to the activation of TRL-1 and TRL-3 signals, facilitating the transfer of IL-17 from the blood to the intestine (36). During the progression of MASLD, mature adipocytes release chemokines, such as *CCL20*, and other soluble mediators, stimulating lymphocyte migration in adipose tissue through CCR6, resulting in obesity-related inflammation, such as insulin resistance, and the occurrence of MASLD (37). Furthermore, high *CCL20* expression in patients with MASLD fibrosis contributed to the development of cholangitis and hepatocellular carcinoma (38). Additionally, *CCL20* can induce cell migration and epithelial-mesenchymal transition (EMT) through the PI3K/AKT and Wnt/β-catenin pathways, thereby promoting tumor growth and predicting low survival rates and tumor recurrence in hepatocellular carcinoma patients (39).

*THY1* is a glycosylphosphatidylinositol-anchored protein weighing–25-37 kDa, known for its high expression on the surface of thymocytes and peripheral T cells. Co-stimulatory bone marrow-derived dendritic cells (BMDCs) induce T cell proliferation through crosslinking of Thy-1 molecules using an anti-Thy-1 monoclonal antibody (mAb; clone G7), which inhibits mitosis (40). Furlong et al. reported that THY-1 signaling facilitates the differentiation of CD4 + T cells into subsets TH1, TH2, and TH17 (41). In a non-polarizing environment, this signal specifically induces the synthesis of IL17A by TH17 cells, promoting immune responses. In a study that employed single-cell sequencing of colonic mesenchymal cells from five patients with IBD, *CD90* enrichment was observed in four clusters of colonic stromal fibroblasts (S1-S4), myofibroblasts, and two pericellular clusters (42). Single-cell sequencing analysis of Crohn’s disease by Martin et al. revealed the presence of an activated fibroblast population. These fibroblasts exhibit high expression levels of *CD90* and PDPN (podoplanin), and the enrichment of this characteristic is associated with resistance to antitumor necrosis factor treatment (43). Although further investigation is required to understand the role of *CD90* fibroblasts and their associated signals in UC, previous studies and our findings support the involvement of *THY1* in immune-related inflammatory diseases. Recently, *THY1* research has gained substantial attention in the field of MASLD. Prior transcriptomic analysis of MASLD patients has revealed that *THY1* is one of the differentially expressed genes linked to the progression from MASLD to NASH and fibrosis. *THY1* is an important biochemical marker for assessing fibrosis progression in MASLD (44). Furthermore, Gao et al. demonstrated an upward trend in *THY1* expression as MASLD progressed, specifically reflecting the severity of fibrosis (AUC=0.74) (45). This observation was subsequently confirmed by Sanchez et al. (46). Collectively, these studies suggest that *THY1*-mediated immune responses and fibrotic mechanisms may represent shared factors that contribute to the co-occurrence of UC and MASLD.

*CD2*, a glycoprotein of the immunoglobulin superfamily, is located on the exterior of T and NK cells. It predominantly attaches to lymphocyte-associated antigen 3 (LFA3, also known as *CD58*) and plays a vital role in establishing and organizing immune synapses between T cells and antigen-presenting cells (47). Previous studies have demonstrated that soluble *CD58* can block the interaction of *CD2/CD58*, thereby playing a therapeutic role in inflammation and autoimmune diseases. The study by Hoffmann et al. has observed a significant decrease in soluble *CD58* in IBD patients, and the levels of *CD58* are significantly are significantly negatively correlated with ESR and disease activity. (48). Recent studies in transgenic mice expressing human *CD219* have demonstrated that treatment with the anti-human *CD2* monoclonal antibody CB.2 in a metastatic colitis model attenuated intestinal inflammation and extended survival, underscoring the substantial role of *CD2* in the onset and progression of UC (49). Although studies on *CD2* in MASLD are limited, enhanced *CD58* expression has been found to correlate with the degree of hepatocellular injury in patients with chronic HBV infection. This could be attributed to its binding to *CD2*, which accelerates the activation of T and NK cells, thus intensifying the cellular immune response to eliminate HBV and potentially leading to liver tissue damage (50). Moreover, in hepatitis C, increased CD2-associated proteins have been demonstrated to influence lipid metabolism through the IRS1-Akt-AMPK-HSL pathway, causing deactivation of hormone-sensitive lipase and consequent augmentation of lipid droplet accumulation, ultimately promoting steatosis (51). We postulate that the co-stimulatory signaling of *CD2-CD58*, inducing T cell activation and hormone-sensitive lipase inactivation, might constitute one of the potential mechanisms contributing to the comorbidity of UC and MASLD.

In this study, we successfully identified and validated four key genes expressed in UC patients with concurrent MASLD, demonstrating their potential involvement in chemokine signaling pathways, neutrophil activation, and focal adhesion functions. Nevertheless, this study has several limitations. First, the lack of available sequencing data on UC patients with concurrent MASLD in the GEO database restricted our ability to explore the differential gene expression between UC and UC with MASLD. Additionally, the acquisition of clinical liver tissue samples poses challenges, necessitating validation of our findings solely using intestinal tissue samples from patients. Future studies should prioritize expanding the sample size and conducting comprehensive validations across multiple tissue types. Furthermore, the absence of additional molecular experiments or animal studies limits our understanding of the mechanistic roles of core genes in the relationship between UC and MASLD. Such investigations will provide a better understanding of this association.

In conclusion, this study identified four genes (*CXCR4, CCL20, THY1*, and *CD2*) associated with MASLD as potential diagnostic markers for UC. These findings suggest that UC may enhance the progression of MASLD through the immune and inflammatory pathways. Additionally, the study revealed significant correlations between the diagnostic markers of UC and MASLD, immune cell infiltration, and specific biochemical indicators in patients with UC. Overall, this study identifies potential biomarkers and treatment options for patients with UC and MASLD.

## Data availability statement

The datasets presented in this study can be found in online repositories. The names of the repository/repositories and accession number(s) can be found in the article/**Supplementary Material**.

## Ethics statement

The studies involving humans were approved by Institutional Review Board of Renmin Hospital of Wuhan University. The studies were conducted in accordance with the local legislation and institutional requirements. Written informed consent for participation was not required from the participants or the participants’ legal guardians/next of kin because We only used patient tissue paraffin blocks for analysis in our study.

## Author contributions

YL: Writing – original draft. JL: Writing – original draft, Conceptualization, Software. ST: Data curation, Methodology, Writing – review & editing. QL: Supervision, Writing – original draft. ZS: Visualization, Writing – review & editing. CL: Supervision, Writing – review & editing, Software. WD: Writing – original draft, Writing – review & editing.
