# Supplementary material for: Identification and validation of hub genes expressed in ulcerative colitis with metabolic dysfunction-associated steatotic liver disease
Source: Front Immunol. 2024 Mar 14;15:1357632. doi: 10.3389/fimmu.2024.1357632 (PMC10972886; doi:10.3389/fimmu.2024.1357632)
Supplement: Supplementary file 1 [file DataSheet_1.docx]

**Identification and validation of co-morbid genes in inflammatory bowel disease combined with non-alcoholic liver disease**

**supplementary material**

**Supplementary Table 1**. Details of samples and genes of all datasets in this study.

| Data sets | PMID | Number of genes | Healthy samples | Patients’ samples |
| --- | --- | --- | --- | --- |
| GSE75214 | PMID:28885228 | 20001 | 11 | 97 UC samples |
| GSE151158 | PMID:32730345 | 618 | 21 | 40 NFALD samples |
| GSE87466 | PMID:29401083 | 21597 | 21 | 87 UC samples |
| GSE33814 | PMID:23071592 | 19419 | 13 | 31 NFALD samples |

**Supplementary Table 2:** The differences between the GEO cohort and the hospital cohort in terms of age and gender

| **Variables** | **GEO Cohort**  **(n=61)** | **Hospital Cohort**  **(n=59)** | **P** |
| --- | --- | --- | --- |
| **Age** | 45.0(33.0, 53.0) | 47.0(36.0, 55.5) | 0.399 |
| **Gender** |  |  | 0.101 |
| Male | 25(41.0) | 33(55.9) |  |
| Female | 36(59.0) | 26(44.1) |  |

**Abbreviation:** GEO**:** Gene Expression Omnibus

**Supplementary Table 3**. The clinical information for all patients in this study.

| **Variables** | **UC**  (n=42) | **MASLD**  (n=7) | **HC**  (n=10) | **P** |
| --- | --- | --- | --- | --- |
| **Age** | 48.5(33.5, 59.0) | 46(38, 51.5) | 45(38.5,47) | 0.660 |
| **Gender** |  |  |  | 0.954 |
| male | 25 | 4 | 4 |  |
| Female | 17 | 3 | 6 |  |
| **Severity** |  |  |  | 0.698 |
| mild | 10 (23.8%) | 1 (14.3%) | - |  |
| moderate | 17 (40.5%) | 4 (57.1%) | - |  |
| Severe | 15 (35.7%) | 2 (28.6%) | - |  |
| **Duration of hospitalization** | 6.0 (4.0 to 8.0) | 4.0 (4.0 to 6.0) | - | 0.321 |
| **Location of lesion** |  |  |  | 0.804 |
| E1 | 3 (7.1%) | 1 (14.3%) | - |  |
| E2 | 18 (42.9%) | 3 (42.9%) | - |  |
| E3 | 21 (50%) | 3 (42.9%) | - |  |
| **Treatment** |  |  |  | 0.774 |
| Symptomatic treatment | 19 (45.2%) | 3 (42.9%) | - |  |
| 5-ASA | 18 (42.9%) | 4 (57.1%) | - |  |
| Steroids | 2 (4.8%) | 0 (0%) | - |  |
| Biological agents | 3 (7.1%) | 0 (0%) | - |  |
| **ESR (mm/h)** | 14.0 (8.0 to 20.0) | 14.0 (5.0 to 21.5) | - | 0.808 |
| **ALT (u/L)** | 14.0 (10.0 to 18.0) | 22.0 (16.5 to 43.0) | 12.0 (9.0 to15.0) | 0.025 |
| **AST(u/L)** | 17.5 (14.0 to 23.0) | 21.0 (18.0 to 29.0) | 15.5 (12.0 to 20.0) | 0.115 |
| **Glucose (mmol/L)** | 5.5 (4.5 to 6.3) | 5.7 (4.5 to 6.4) | 5.1 (4.2 to 6.0) | 0.857 |
| **Triglycerides (mmol/L)** | 0.9 (0.7 to 1.2) | 2.0 (1.5 to 3.8) | - | 0.002 |
| **Cholesterol (mmol/L)** | 3.7 ± 0.8 | 4.0 ± 1.3 | - | 0.639 |
| **HDL-C(mmol/L)** | 1.1±0.3 | 1.0±0.3 | - | 0.210 |
| **LDL-C(mmol/L)** | 2.2±0.6 | 2.8 ± 1.3 | - | 0.289 |
| **BMI (kg/m^2^)** | 20.7 ± 1.7 | 21.2 ± 1.9 | 20.2 ± 1.5 | 0.515 |
| **Complications** |  |  |  |  |
| Dyslipidemia | 6 (14.3%) | 5 (71.4%) | 0 | <0.001 |
| Hypertriglyceridemia | 3 (7.1%) | 2 (28.6%) | 0 | 0.083 |
| Hypertension | 4 (9.5%) | 1 (14.3%) | 0 | 0.700 |
| Diabetes Mellitus | 7 (16.7%) | 2 (28.6%) | 0 | 0.451 |
| Prediabetes | 3 (7.1%) | 2 (28.6%) | 1(10%) | 0.700 |
| Overweight | 5 (11.9%) | 2 (28.6%) | 1(10%) | 0.243 |

**Note:**

**Location of lesion:** The extent of UC lesions is mainly defined according to the Montreal classification. **E1** represents ulcerative proctitis, with inflammation limited to the rectum; **E2** represents left-sided colitis, with lesions extending to the left half of the colon; and **E3** represents extensive colitis, with lesions involving the splenic flexure to the entire colon.

**Treatment:** Symptomatic treatment primarily includes antibiotics, mucosal protectants, and traditional Chinese herb; 5-ASA treatment primarily includes mesalamine and sulfasalazine; Steroids treatment primarily includes prednisone and dexamethasone; Biological agent treatment primarily includes vedolizumab. When merging or recording for UC patients who have been treated with multiple medications, the highest tier medication is selected (Biological agents> Immunosuppressants>5-ASA> Symptomatic treatment).

**Diagnostic criteria**

**Dyslipidemia**: Dyslipidemia is defined as meeting at least one of the following criteria: TG level ≥ 1.70 mmol/L; TC level ≥ 5.18 mmol/L; LDL-C level ≥ 3.37 mmol/L; HDL-C level < 1.04 mmol/L.

**Hypertriglyceridemia**: Hypertriglyceridemia is defined as TG level ≥ 1.70 mmol/L.

**Hypertension**: Hypertension is defined as having a systolic blood pressure ≥ 140 mmHg and/or a diastolic blood pressure ≥ 90 mmHg.

**Diabetes Mellitus**: Diabetes is defined as having a fasting blood glucose level ≥ 7 mmol/L or a postprandial or random blood glucose level ≥ 11.1 mmol/L, accompanied by typical symptoms of diabetes.

**Prediabetes**: Prediabetes is defined as having a fasting blood glucose level ≥ 6.1 mmol/L and ≤ 7.0 mmol/L, or a 2-hour postprandial blood glucose level ≥ 7.8 mmol/L and ≤ 11.1 mmol/L.

**Overweight:** Overweight is defined as BMl≥23 kg/m^2^.

**Abbreviation**: UC: ulcerative colitis; MASLD, metabolic dysfunction-associated steatotic liver disease; HC: healthy control; 5-ASA: 5-aminosalicylic acid; ESR: erythrocyte sedimentation Rate. HDL-C: High density lipoprotein cholesterol. LDL-C: Low-Density Lipoprotein Cholesterol. BMI: Body Mass Index.

**Supplementary Table 4**: The p-values, q-values, and NES of the top 5 gene sets related to hub genes in GSE75214.

| Gene-sets | CXCR4 | | | CD2 | | | THY1 | | | CCL20 | | |
| --- | --- | --- | --- | --- | --- | --- | --- | --- | --- | --- | --- | --- |
|  | p-val | FDR q-val | NES | p-val | FDR q-val | NES | p-val | FDR  q-val | NES | p-val | FDR  q-val | NES |
| Allograft rejection | <0.001 | <0.001 | 2.404 | <0.001 | <0.001 | 2.329 | <0.001 | 0.001 | 2.165 | <0.001 | 0.004 | 2.011 |
| Graft-versus-host-disease | <0.001 | <0.001 | 2.403 | <0.001 | <0.001 | 2.310 | <0.001 | 0.001 | 2.228 | 0.008 | 0.035 | 1.719 |
| leishmaniasis | <0.001 | <0.001 | 2.566 | <0.001 | <0.001 | 2.600 | <0.001 | 0.001 | 2.440 | <0.001 | 0.004 | 1.929 |
| malaria | <0.001 | <0.001 | 2.615 | <0.001 | <0.001 | 2.544 | <0.001 | 0.001 | 2.424 | <0.001 | 0.004 | 1.912 |
| Primary immunodeficiency | <0.001 | <0.001 | 2.328 | <0.001 | <0.001 | 2.359 | <0.001 | 0.001 | 2.118 | <0.001 | 0.004 | 1.911 |

**Abbreviation:** NES: normalized enrichment scores

| Gene-sets | CXCR4 | | | CD2 | | | THY1 | | | CCL20 | | |
| --- | --- | --- | --- | --- | --- | --- | --- | --- | --- | --- | --- | --- |
|  | p-val | FDR  q-val | NES | p-val | FDR  q-val | NES | p-val | FDR  q-val | NES | p-val | FDR q-val | NES |
| Rheumatoid arthritis | <0.001 | 0.012 | 1.657 | 0.029 | 0.210 | 1.341 | 0.021 | 0.197 | 1.430 | 0.007 | 0.181 | 1.481 |
| Asthma | - | - | - | 0.005 | 0.082 | 1.541 | 0.003 | 0.104 | 1.656 | 0.005 | 0.082 | 1.541 |
| Graft-versus-host disease | 0.010 | 0.195 | 1.487 | 0.004 | 0.082 | 1.500 | 0.032 | 0.245 | 1.427 | - | - | - |
| Primary immunodeficiency | 0.029 | 0.273 | 1.410 | 0.007 | 0.082 | 1.485 | 0.017 | 0.174 | 1.493 | 0.009 | 0.181 | 1.507 |
| Allograft rejection | 0.010 | 0.195 | 1.487 | 0.007 | 0.082 | 1.478 | 0.005 | 0.128 | 1.574 | 0.023 | 0.208 | 1.424 |
| Type I diabetes mellitus | 0.011 | 0.195 | 1.487 | 0.012 | 0.115 | 1.441 | 0.007 | 0.128 | 1.573 | 0.022 | 0.208 | 1.435 |

**Supplementary Table 5**: The p-values, q-values, and NES of the top 5 gene sets related to hub genes in GSE151158

**Abbreviation:** NES: normalized enrichment scores

**Supplementary Table 6**: The IRS score of core genes among different UC disease activity groups.

| Genes | Mild  （N=10） | Moderate（N=17） | Severe  （N=15） | P value |
| --- | --- | --- | --- | --- |
| CXCR4, median (IQR) | 5.5 (4.2, 8.0) | 7.0 (6.0, 8.0) | 7.0 (6.0, 8.5) | 0.438 |
| THY1, median (IQR) | 4.0 (4.0, 5.0) | 5.0 (4.0, 6.0) | 4.0 (4.0, 5.0) | 0.837 |
| CCL20, median (IQR) | 7.0 (3.75, 8.0) | 7.5 (7.0, 8.0) | 7.0(6.0, 7.5) | 0.517 |
| CD2, median (IQR) | 3.5 (3.0, 4.8) | 4.0 (2.0, 5.0) | 4.0 (3.0, 5.0) | 0.771 |
| CD86, median (IQR) | 3.0 (2.0, 3.8) | 4.0 (3.0, 4.0) | 4.0(3.0, 5.0) | 0.107 |

**
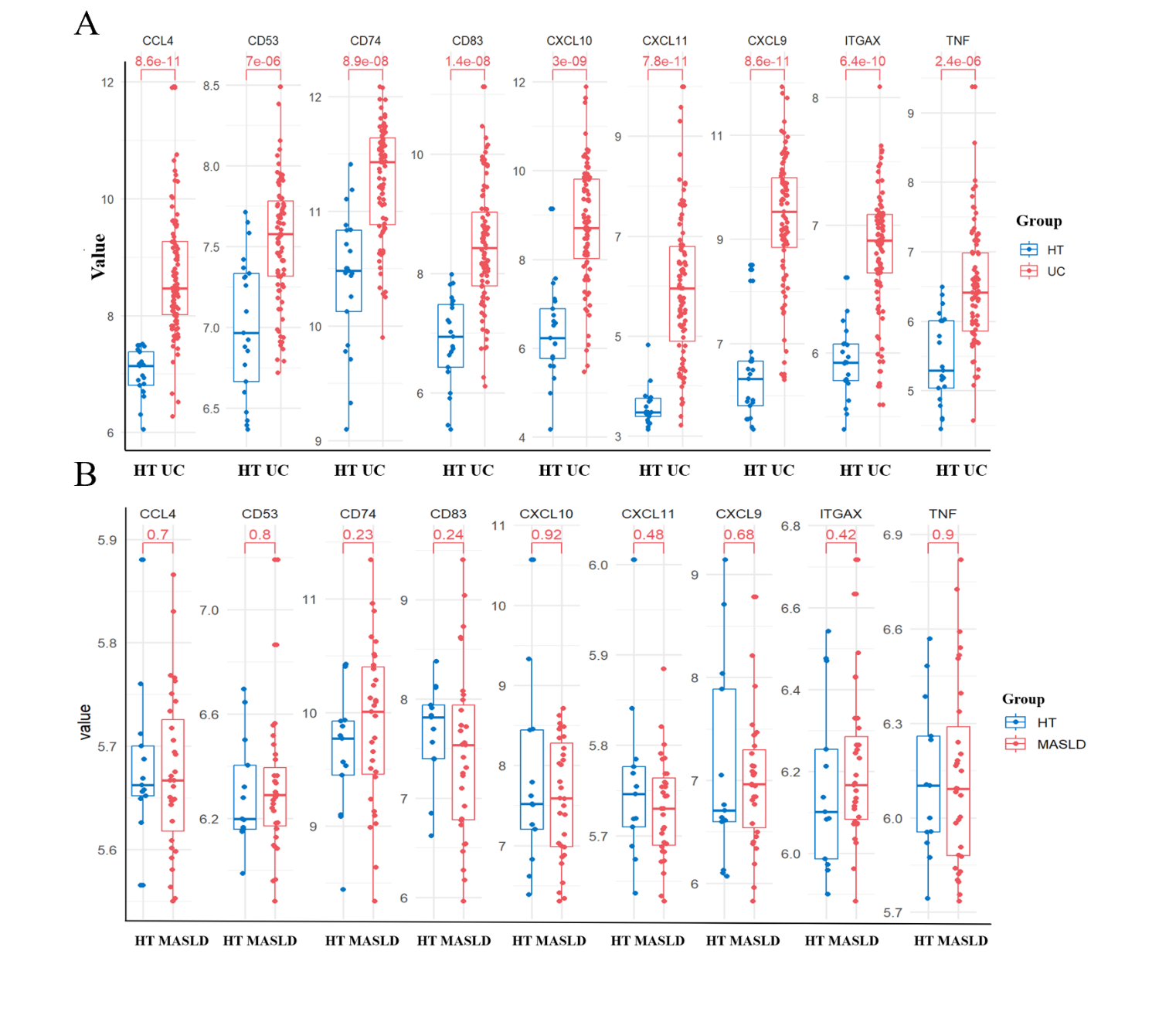
Supplementary Figure 1:** Expression of the candidate hub genes in validation dataset GSE87466 **(A)** and GSE33814 **(B).**

**Abbreviation:** UC: ulcerative colitis; MASLD, metabolic dysfunction-associated steatotic liver disease

**Supplementary Figure 2:** A merged enrichment plot of CXCR4、THY1、CCL20 and CD2 from gene set enrichment analysis in dataset GSE75214**(A)** and GSE151158**(B)**

**
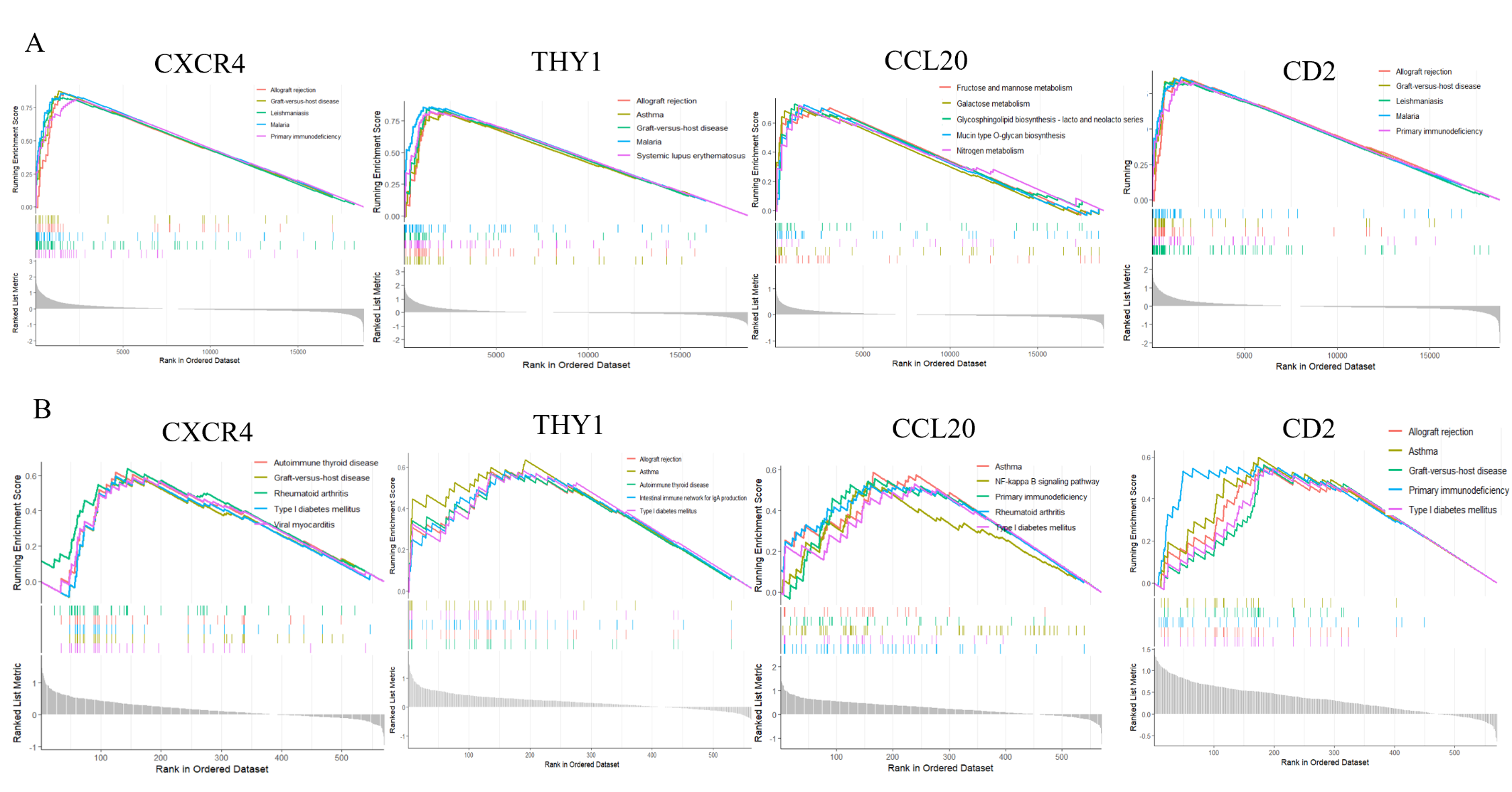
**

**Supplementary Figure 3:** A merged enrichment plot of CXCR4、THY1、CCL20 and CD2 from gene set enrichment analysis in validation dataset GSE87466**(A)** and GSE33814**(B)**


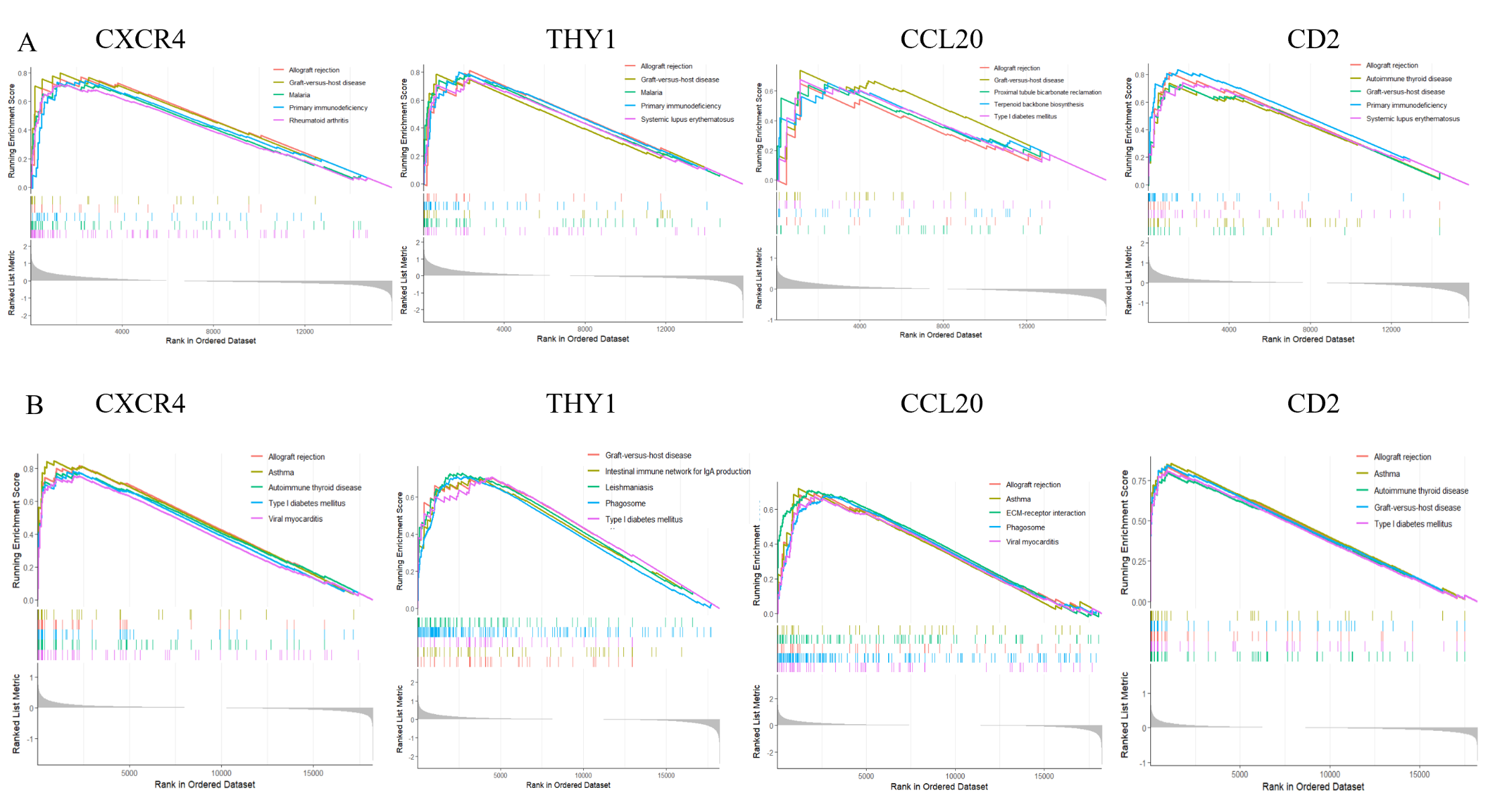


**Supplementary Figure 4:** The Representative immunohistochemical images of CXCR4**(A)**, CCL20**(B)**, THY1**(C)**, CD2**(D)** and CD86**(E)** among different UC disease activity groups.


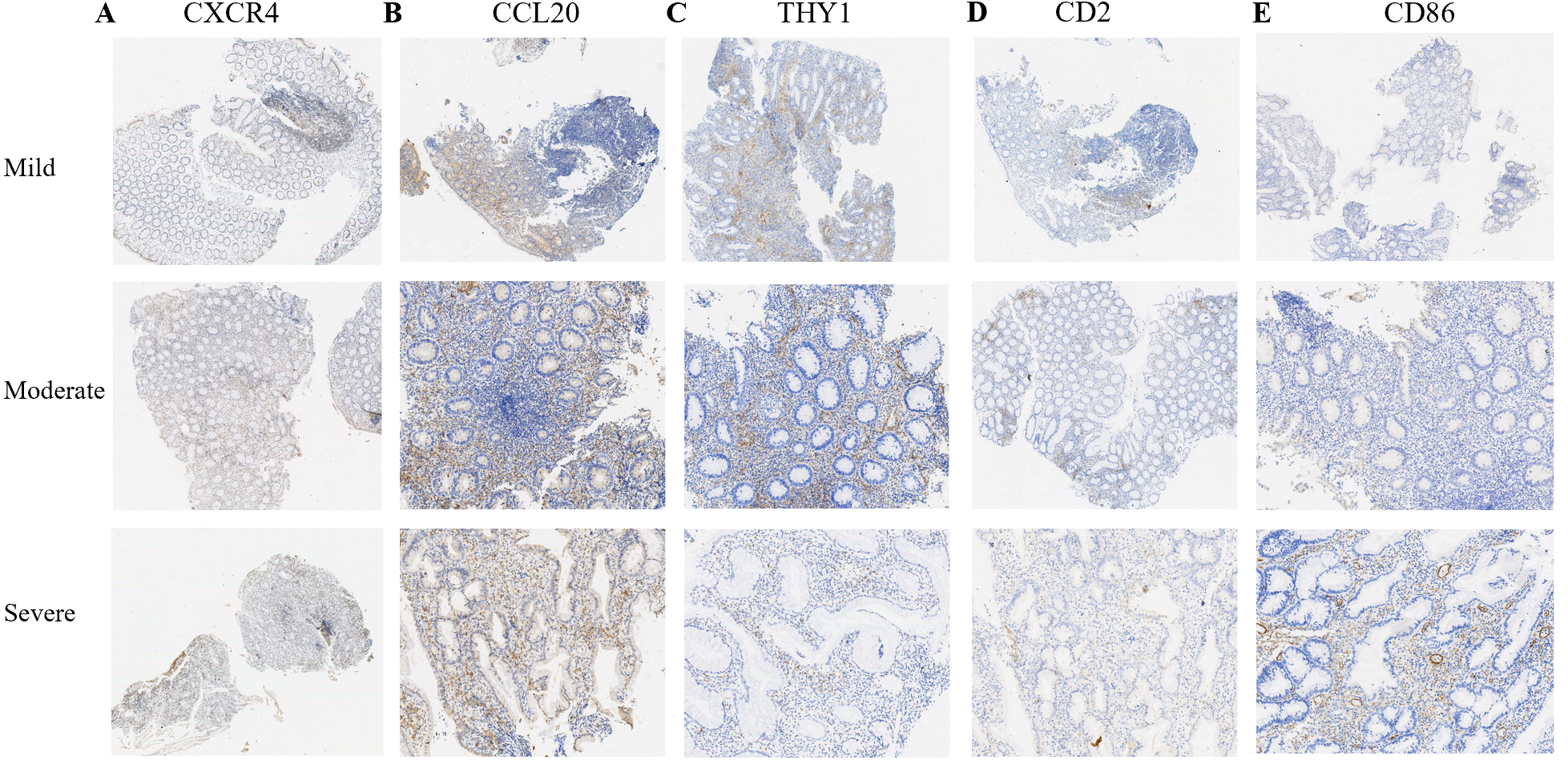


**Abbreviation:** UC: ulcerative colitis

**Supplementary Figure 5:** Scatterplot of correlation between total core gene expression score and albumin**(A)**、Prothrombin time**(B)** and INR**(C)**. INR：PT International Normalized.


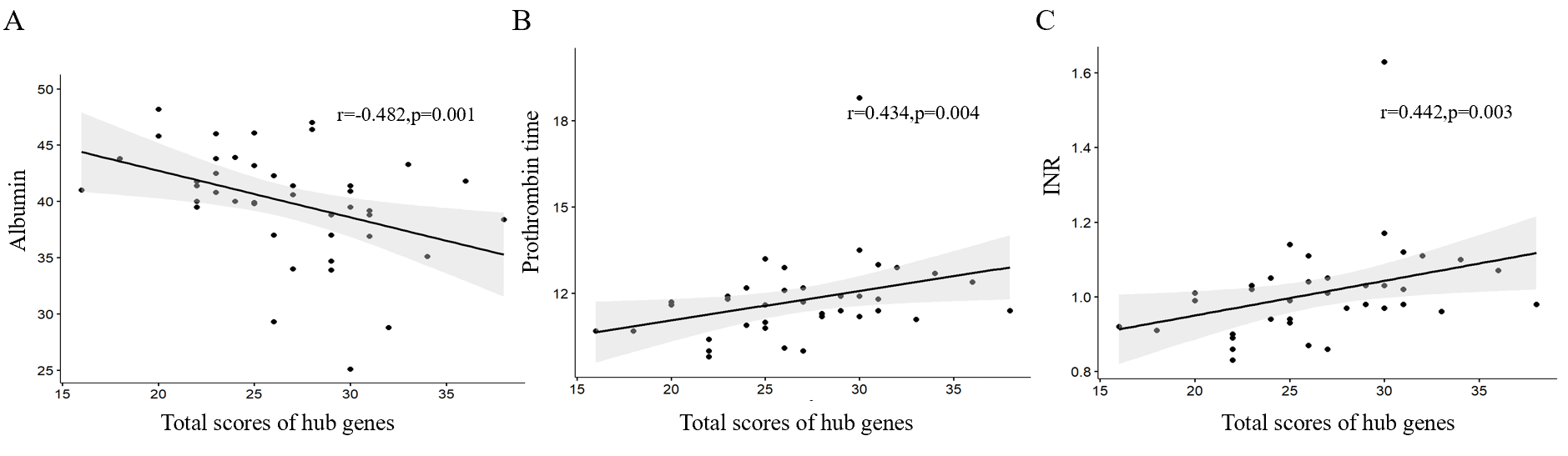


**Abbreviation:** INR：PT International Normalized.
